# Breaking the dichotomy of HER2: highlighting the flaws of simplifying variables

**DOI:** 10.3389/fonc.2025.1494417

**Published:** 2025-05-09

**Authors:** Jacob Pozin, Reid Shaw, Jennifer Weiss

**Affiliations:** Department of Hematology/Oncology, Loyola University Medical Center, Maywood, IL, United States

**Keywords:** HER2, breast cancer, trastuzumab, IHC, dichotomization, mass spectrometry

Forecasting and modeling is an inherently imprecise practice. However, the goal is to make predictions that are useful and as accurate as possible. Dichotomization, which is common in medicine, is universally considered a poor practice in modeling. Dichotomization leads to the loss of information, loss of effect size and power, spurious statistical significance, potential to overlook non-linear relationships, and loss of measurement reliability. (1–3) Despite this, it is perceived to ease interpretability, simplify analysis by reducing complexity, or allow for comparisons between multiple groups. However, few physicians would argue that a hemoglobin A1c of 6.5% is equivalent to 12% – despite both values meeting the criteria for diabetes. (4) When a continuous variable is dichotomized, the information contained in the specific values vanishes. In contrast, an individual with an A1c of 6.4% is unlikely to have significantly different outcomes than that of an individual with an A1c of 6.5%. However, with dichotomization, they are now viewed as distinct entities – decreasing the effect size and power. Although clinicians are unlikely to treat these scenarios similarly, problems arise when the dichotomized variable is less well understood and reporting on disease status is not standardized – as is the case of HER2-low in breast cancer.

Amplification of the HER2 gene is present in approximately 15% of early-stage breast cancer and is associated with an aggressive phenotype and increased risk of disease recurrence. Fortunately, there are HER2-targeting agents, such as trastuzumab, a humanized monoclonal antibody that improve overall survival in this patient cohort. (5) This landmark randomized control trial (RCT) used immunohistochemistry (IHC) to identify patients who may benefit from trastuzumab. (6) IHC separates HER2 into semi-quantitative categories: positive (3+), equivocal (2+), and negative (1+ and 0). (7) Although, HER2-positive tumors are most likely to respond to HER2-targeting therapy, the *post-hoc* analysis demonstrated that certain HER2-negative patients derived benefit from trastuzumab – opening the door for a new subset of patients termed HER2-low. (8) HER2-low is defined as IHC 1+ or 2+ with no amplification of HER2 by fluorescent *in situ* hybridization, a costly, but more accurate method of assessing HER2 status. However, when assessed in an RCT, trastuzumab failed to demonstrate a survival benefit in HER2-low breast cancer. (9) In support of these findings, multiple studies demonstrate that the HER2-low classification is not a reproducibly defined subtype of breast cancer with any distinct prognostic implication. (10–14) Despite these contradicting results, the drug-antibody conjugate, trastuzumab deruxtecan, showed improved overall survival in the HER2-low cohort. (15) It even demonstrated an objective response rate of 30% in HER2-negative (IHC 0) metastatic breast cancer. (16) Although the debate surrounding HER2-low as a distinct entity continues, the 2023 ASCO-College of American Pathologists encourages the reporting comment of IHC 0 versus 1+ to ensure identification of patients eligible for trastuzumab deruxtecan. These guidelines indicate the differentiation in IHC classification of 0 and 1+ is based on the detection of faint or barely perceptible incomplete membrane staining of amplified HER-2 in less or greater than 10% of tumor cells, respectively. (17) However, one survey of the College of American Pathologists shows that approximately 20% of cases read by laboratories generate results with less than 70% concordance for IHC HER2 score 0 versus 1+ (10). Despite this discrepancy, DESTINY-Breast06 is currently investigating trastuzumab deruxtecan in HER2-low and HER2-ultralow (IHC >0 <1+ expression). The results from the DESTINY-Breast06 trial presented at ASCO 2024 revealed that both HER2 low and ultralow cancers had similarly increased PFS and objective response rates (18). Although the DESTINY studies utilize a novel IHC assay to assess HER2 status, it is not clear if this test has less discordance than prior assays. To address this problem, quantitative measurements of HER2 are being developed that utilize mass spectrometry. (19) These methods of multiple reaction monitoring mass-spectrometry, which evaluate formalin-fixed-paraffin-embedded breast cancer tissues, have been found to both offer numerical quantification of HER2 levels that correlates well with established IHC classification, even at low HER2 expression levels (20, 21), as well as possibly distinguish between HER2-positive and HER2-negative samples with high specificity and sensitivity (22). Mass spectrometry identification of HER2 levels has also been shown to serve as a prognostic method in determining which patients will benefit from trastuzumab treatment. Specifically, higher HER2 levels (threshold >2200 amol/µg) were significantly associated with longer disease-free survival and overall survival in an adjuvant setting, as well longer overall survival in a metastatic setting (23). However, for quantitative HER2 methods to have clinical utility, they would need to be validated in trials studying whether they are predictive of benefit from HER2 targeted therapies in patients with HER2-positive, HER2-low, and HER2-ultralow breast cancer (24). Currently, we do not report ultralow HER2, however with HER2-ultralow showing clinical significance, it likely will change how we report HER2 status. For this reason, we need more standardized procedures, guidelines, and specialized training for pathologists in order to more accurately assess and report HER2-low and ultralow scores (25). Potentially, artificial intelligence IHC quantifier software will be used to consistently and accurately score HER2 expression (26).

Although the aim of this essay is not to settle the debate surrounding the legitimacy of HER2-low or HER2-ultralow as distinct biological entities, there are more appropriate and statistically rigorous means of evaluating HER2-targeted therapies. As evidenced by multiple prior studies, the answer is not to arbitrarily bin variables. Rather, HER2 expression should be kept as a continuous variable, with different treatment modalities based on a spectrum of HER2 expression. Treating HER2 as a continuous variable will likely decrease the number of patients needed in a clinical trial. (27) Furthermore, instead of spurious odds ratios determined by arbitrary cut points, the data may be presented as a simple line graph and confidence bands. (27) As the landscape continues to develop surrounding the clinical significant of HER2 status, newer, more standardized modalities to accurately evaluate and report its expression are increasingly needed to help individualize patient care and improve outcomes.
